# Editorial: Community series in the consequences of COVID-19 on the mental well-being of parents, children and adolescents, volume II

**DOI:** 10.3389/fpsyt.2023.1249748

**Published:** 2023-07-11

**Authors:** Sevtap Gurdal, Maria Bacikova-Sleskova, Sabina Kapetanovic, Soly I. Erlandsson, Emma Sorbring

**Affiliations:** ^1^Department of Social and Behavioural Studies, University West, Trollhättan, Sweden; ^2^University of Pavol Jozef Šafárik, Košice, Slovakia

**Keywords:** COVID-19, emotional distress, education, social distancing, isolation, family

While the COVID-19 pandemic affected all people in some way, some were affected more than others. Society was forced to act quickly and adapt to new restrictions and rules in their everyday lives. Social distancing was adopted, people were forced into lockdowns and quarantines while schools and working places were closed and people made to work from home.

This second edition of the Research Topic aims to extend knowledge of the consequences of the COVID-19 pandemic and widen perspectives of the impact that it has had worldwide. The nine included papers come from different countries (Thailand, China, Malawi, Slovakia, Italy, and Germany) and cover various topics related to the pandemic. Four of the papers focus on data from adolescents and their mental health. Three papers use data from parents, how parents perceive changes in family dynamics, their own mental health as well as how they coped with the pandemic while the final two focus on instrument validation.

Adolescents are a vulnerable group that had to make great changes to their lives during the pandemic. Their lives changed significantly due to lockdown and not being able to meet friends. Guo et al. explored how adolescents aged 12–18 perceived lives during lockdown in 2022. A sample of 1,065 Chinese middle and high-school students filled in questionnaires to examine the association between social support, rumination, sleep quality and negative emotional states (e.g., depression, anxiety, and stress). The findings from this study showed that bad sleep quality and less social support is negatively associated with negative emotional states but positively correlated with rumination. These results highlight that social support for adolescents is important in decreasing negative emotions such as depression, anxiety and stress.

A longitudinal study from Germany by Güzelsoy et al. aimed to identify the risk and protective factors of depressive symptoms and anxiety in children and adolescents during the pandemic. In their study, the authors used data from about 800 adolescents and their parents from a baseline and at a 6-month follow up. The results showed that cross-sectionally, several family and school factors were associated with adolescent depression and anxiety. However, longitudinally only parental depressive symptoms were risk factors for depression and anxiety 6 month later.

Studies from different parts of the world are very important in understanding the impact of COVID-19 worldwide. A study carried out by Mmanga et al. in Malawi has shown results that are consistent with those from economically more advantaged countries. The authors used quantitative and qualitative methods to study depression and anxiety among adolescents and reported quite a high prevalence of depressive symptoms and anxiety. They also stress the need to improve the knowledge of Malawi adolescents in terms of the virus, vaccinations and mental health related topics.

Wang et al. provide a complex picture about depression and anxiety among children and adolescents pre- and post-COVID-19 in their meta-analysis. A selection process identified seven longitudinal studies (four from the USA, and one from each of the following countries: Canada, Greece, and Australia) that had studied depression and anxiety before and after the pandemic. The study showed a significant deterioration of child and adolescent mental health in the post COVID-19 period.

Parents have been affected by the pandemic in different ways and have even been seen as the most vulnerable group in some way (Vargová et al.). In Slovakia, the importance of parents' mental health was the focus of a study where a sample of 363 parents participated in four waves of data collection over one and a half years. The results showed a small change in depression and anxiety but higher changes in COVID-related stress and anxiety. There was also a stability that grew over time in that stress or anxiety did not increase but stayed the same or even decreased. This could be explained by parents adapting to the situation that the pandemic created. The authors highlight that the parents' wellbeing in a crisis like a pandemic is important since parents' wellbeing can have a spillover effect on their children's wellbeing.

Limsuwan et al. examined the effects of the pandemic on family wellbeing in Thailand. They used a cross-sectional study to learn more about the changes in family functioning and happiness. A questionnaire was distributed online and on Facebook with 485 participants answering questions about perceived family happiness pre-pandemic and post-pandemic. The majority (90.9%) were women. The score showed a slight decrease in the post-pandemic score. Moreover, general family functioning, strength and communication were significantly lower after 1 year of the pandemic. On the other hand, the results showed a lower level of verbal and physical violence. The strongest association to the change of family functioning was the change in family happiness which decreased during the pandemic.

Another study from China conducted by Ma et al. looked at how the pandemic affected children with intellectual disabilities during lockdown. Furthermore, it also explored the parent coping strategies. There were 457 parents recruited for the study who had at least one child between 12 and 18 with an intellectual disability. The analyses revealed that children with an intellectual disability had the most positive changes concerning sleep, diet and communication which were reported to function better during the pandemic. However, hyperactivity and inappropriate language did not improve. Parents used different coping strategies staying at home with their children, with the most popular ones being diversion (i.e., watching TV, eating, or playing with a mobile phone). However, it is not clear which coping strategy worked best and more research is needed to get a clear answer.

Valid instruments need to be implemented in research in order to be able to study the impact of the pandemic. Within this Research Topic, there are two validation papers included. The first paper by Yang et al. aims to study the factor structure and measurement invariance of the COVID-19 Phobia Scale in Chinese adolescents with depressive symptoms. This has been explored among nearly 2,000 adolescents. The instrument showed a stable four-factor structure (psycho-somatic factor, psychological, economic and social factors) and had good reliability and validity in the sample.

The second validation study carried out by De Stefano et al. aimed to explore the psychometric properties of the Children and Adolescents Psychological Distress Scale CAPDS-10. The new scale was developed to study distress during the COVID-19 pandemic with nearly 3,500 French children and adolescents filling in the questionnaire. The 10-item scale measures psychological distress over the most recent 2-weeks and has robust unidimensional structure and good psychometric properties. As the authors state, it could be used in crisis or prevention contexts in the general population or in clinical settings.

## Author contributions

All authors listed have made a substantial, direct, and intellectual contribution to the work and approved it for publication.

